# An *AARS* variant as the likely cause of Swedish type hereditary diffuse leukoencephalopathy with spheroids

**DOI:** 10.1186/s40478-019-0843-y

**Published:** 2019-11-27

**Authors:** Christina Sundal, Susana Carmona, Maria Yhr, Odd Almström, Maria Ljungberg, John Hardy, Carola Hedberg-Oldfors, Åsa Fred, José Brás, Anders Oldfors, Oluf Andersen, Rita Guerreiro

**Affiliations:** 1 Department of Clinical Neurology, Institute of Neuroscience and Physiology, the Sahlgrenska Academy, University of Gothenburg, Gröna Stråket 11, 3rd floor, Sahlgrenska University Hospital, 413 45 Göteborg, Sweden; 2 Center for Neurodegenerative Science, Van Andel Institute, 333 Bostwick Ave. N.E, Grand Rapids, MI 49503-2518 USA; 3 Department of Laboratory Medicine, Institute of Biomedicine, the Sahlgrenska Academy, University of Gothenburg, Göteborg, Sweden; 4 Department of Radiation Physics, Institute of Clinical Sciences, the Sahlgrenska Academy, University of Gothenburg, Göteborg, Sweden; 5 Department of Neurodegenerative Disease, Reta Lila Weston Laboratories, Queen Square Genomics, UCL Dementia Research Institute, London, UK; 6 Department of Pathology, Hospital of Halland, Halmstad, Sweden; 7 Division of Psychiatry and Behavioral Medicine, Michigan State University College of Human Medicine, Grand Rapids, MI USA

**Keywords:** Swedish type hereditary diffuse Leukoencephalopathy with spheroids, HDLS, Alanyl tRNA synthetase, AARS

## Abstract

Swedish type Hereditary Diffuse Leukoencephalopathy with Spheroids (HDLS-S) is a severe adult-onset leukoencephalopathy with the histopathological hallmark of neuraxonal degeneration with spheroids, described in a large family with a dominant inheritance pattern. The initial stage of the disease is dominated by frontal lobe symptoms that develop into a rapidly advancing encephalopathy with pyramidal, deep sensory, extrapyramidal and optic tract symptoms. Median survival is less than 10 years. Recently, pathogenic mutations in *CSF1R* were reported in a clinically and histologically similar leukoencephalopathy segregating in several families. Still, the cause of HDLS-S remained elusive since its initial description in 1984, with no *CSF1R* mutations identified in the family. Here we update the original findings associated with HDLS-S after a systematic and recent assessment of several family members. We also report the results from exome sequencing analyses indicating the p.Cys152Phe variant in the alanyl tRNA synthetase *(AARS)* gene as the probable cause of this disease. The variant affects an amino acid located in the aminoacylation domain of the protein and does not cause differences in splicing or expression in the brain. Brain pathology in one case after 10 years of disease duration showed the end stage of the disease to be characterized by widespread liquefaction of the white matter leaving only some macrophages and glial cells behind the centrifugally progressing front. These results point to *AARS* as a candidate gene for rapidly progressing adult-onset CSF1R-negative leukoencephalopathies.

## Introduction

Adult onset leukodystrophies are a heterogenous group of genetic disorders associated with myelin pathology [13, 21]. However, some entities with striking axonal histopathology were characterized as neuraxonal degenerations. An adolescence-to adult onset leukoencephalopathy with axonal spheroids and glial lipofuscin-like pigment was described [38], later termed Pigmentary Orthochromatic Leukodystrophy (POLD). The histopathological changes of the cerebral aspect of giant axonal neuropathy [26] and Nasu-Hakola disease [11] also include numerous axonal spheroids. In 1984, we described a severe adult onset leukoencephalopathy occurring in a dominant segregation pattern in a large western Swedish family, with histopathology assessment performed in four autopsies available from two generations. The results from this analysis showed numerous axonal spheroids suggestive of primary axonal damage. This disease was termed Hereditary Diffuse Leukoencephalopathy with Spheroids (HDLS) [4]. The homogeneity of the clinical and histopathological features strongly suggested that HDLS was a distinct diagnosis, and it remained a clinical entity [39]. This Swedish kindred was recently re-examined [33].

The genetic study of several US families with at least one neuropathologically confirmed HDLS case, and the expansion of this study to an international consortium, led to the identification of disease causative mutations in the gene coding for the colony stimulating factor 1 receptor (*CSF1R*) [27]. Follow up studies have confirmed this finding with several other causative mutations in the kinase region of this protein now known to cause HDLS [9]. More recently mutations in this gene have also been associated with the development of POLD and the diagnostic term Adult-onset Leukoencephalopathy with Spheroids and Pigmented glia (ALSP) was coined as a unifying identifier for POLD and HDLS, justified by their essentially common neuropathology [1]. However, not all cases of leukoencephalopathy with neuroaxonal spheroids from an international biobank material carried *CSF1R* mutations [9], and until now no locus has been associated with the original Swedish HDLS [4] (HDLS-S).

To identify the genetic cause of disease in this HDLS-S family we performed exome sequencing and followed up the results with Sanger sequencing. Twenty-five family members participated in this study (2 were affected and 23 were healthy).

## Material and methods

**Fig. 1:**
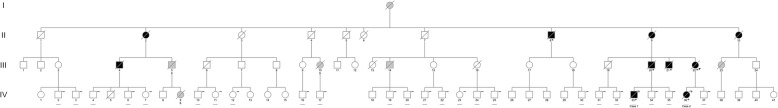
Family pedigree updated from Axelsson et al., 1984 and Sundal et al., 2012a. Black symbols indicate well-documented HDLS-S patients (with available hospital records and examinations in person); grey symbols indicate HDLS patients with more sparse documentation. White symbols indicate unaffected family members. Circles indicate females, squares indicate men. The proband is indicated by an arrowhead. A diagonal line through circles or squares indicates a deceased person. Asterisks (*n* = 6) indicate neuropathological examination demonstrating HDLS histopathology, as previously reported in Axelsson et al., 1984 and Sundal et al., 2012a and in the present case 1. The letter S under the symbols indicates suicide. A black horizontal line below circles or squares in 25 individuals in generation IV, including cases 1 and 2 in the present study, indicates that a standardized neurological examination and/or personal follow-up was performed during 2018. Sanger sequencing results for the presence or absence of the *AARS* p.Cys152Phe variant are shown as ‘+’ and ‘-‘, respectively

All samples included in this study were collected from one large Swedish family originally described in 1984 [4]. The updated family tree is presented in Fig. 1 with four generations represented and including information from the most recent clinical assessments. DNA was extracted using QIAsymphony, following standard procedures. This study was approved by the Research Ethics Committee of Gothenburg (601–05, 1016–12).

### Genome-wide genotyping

Samples from both patients (IV-33 and IV-36) and two unaffected relatives (IV-21 and IV-35) were genotyped using Illumina Infinium technology to identify the presence of any large structural variants (> 50 Kb) and large regions of homozygosity (> 1 Mb). The samples were genotyped using the HumanOmniExpress BeadChip according to manufacturer’s instructions, and data were visualized using the GenomeStudio Data Analysis Software (Illumina Inc.).

### Exome sequencing

Genomic DNA samples from the two patients (IV-33 and IV-36) and two unaffected relatives (IV-21 and IV-35) were subjected to exome sequencing. Capture was performed using Illumina TruSeq Exome kit, and sequencing was done on Illumina’s HiSeq 2000 according to manufacturer’s instructions. After quality filters, reads were aligned to the hg19/GRCh37 reference genome using BWA v0.7.12 [18] and variants were called using GATK best practices v3.3–0 [8, 22].

### In silico analyses

Single nucleotide polymorphisms (SNPs) and insertions and deletions (Indels) were filtered to select rare exonic and splice-site variants predicted as deleterious (more details in the Additional file 1). In parallel we ranked the variants obtained from the exome sequencing and taking into account the diagnosis and family pedigree using Exomiser v7.2.1 [32] with the following parameters: autosomal dominant inheritance pattern, minor allele frequency (MAF) below 0.1%, and OMIM entry for Leukoencephalopathy, diffuse hereditary, with spheroids (**#** 221820). The predicted functional impact of the variants was evaluated using SIFT [30], PolyPhen-2 [2], MutationTaster2 [29] and CADD v1.3 [12] software. ClinVar (http://www.ncbi.nlm.nih.gov/clinvar/) was also used in the variants’ classification process.

### Sanger sequencing

Candidate variants in *AARS* and *ESRP2* were validated and screened in 21 additional family members of generation IV using Sanger sequencing. Regions containing the candidate variants were amplified by polymerase chain reaction (PCR) with Roche FastStart PCR Master Mix (Roche Diagnostics Corp) and sequenced with Applied Biosystems BigDye terminator v3.1 sequencing chemistry in an ABI3730XL genetic analyzer as per manufacturer’s instructions (Applied Biosystems). Primers are available upon request. The sequences were analyzed in Sequencher software v4.2 (Gene Codes) using ENSG00000090861 and ENSG00000103067 as reference sequences for *AARS* and *ESRP2*, respectively.

### Expression analysis

For functional analysis of the missense variant identified in *AARS*, c.455G > T p.(Cys152Phe), total RNA was isolated from a post mortem occipital brain tissue sample stored in RNAlater, using the RNeasy Fibrous Tissue Mini Kit (Qiagen, Valencia, CA). RNA was reverse transcribed with the QuantiTect reverse transcription kit (Qiagen), and *AARS* cDNA was analyzed by PCR with 20, 23 and 27 cycles followed by Sanger sequencing (primers are available upon request).

### Neuropathology analyses

A postmortem examination was performed in case 1 at 57 years of age. The left part of the brain, most of the spinal cord and the left sciatic nerve were fixed in buffered paraformaldehyde. A part of the right half of the brain was frozen and stored at − 80^0^ C and the rest was fixed in buffered glutaraldehyde together with a part of the cervical spinal cord and the right sciatic nerve. Microscopic examination included various parts of the cerebral cortex and underlying white matter, the basal ganglia, thalamus, brain stem, cerebellum, spinal cord and sciatic nerve. Staining of formalin-fixed paraffin embedded sections included hematoxylin-eosin, van Gieson staining, luxol fast blue-cresyl violet, periodic acid and Schiff reagent (PAS) and Berliner blue (iron). Autofluorescence was examined on unstained sections. Immunohistochemistry was performed for neurofilament protein (Dako M0762, 1:400), glial fibrillary acidic protein (GFAP; Dako Z0334, 1:10,000), CD68 (Dako IR613, 1:1) as a marker for macrophages and ubiquitin (Abcam ab134953).

## Results

### Updated information on the HDLS-S family

The ancestor born in 1857 (I:1 in the pedigree) lived on a farm in an isolated area of Western Sweden. She suffered for 32 years from an undefined mental disorder with “epilepsy” and died from “brain hemorrhage”, but no medical records remain [4]. Of 8 HDLS cases in generations II-III, 5 were documented by relevant diagnostic codes or hospital records from regional psychiatric or geriatric institutions, with HDLS confirmed by histopathology in one (II:8). Three siblings in generation III were examined at our Gothenburg neurology or psychiatry university departments with a preliminary hospital diagnosis of presenile dementia. These 3 cases were the first in whom our clinical, genealogical, and neuropathological examinations defined HDLS (III:20, III:21 and III:22). Individual III:22 had consecutive neurological assessments throughout the course of disease. Two family members in generation III and one in generation IV committed suicide during middle age. Even though the documentation associated with these cases was sparse, we believe these suicidal cases could have been associated with incipient HDLS. One individual (III:19) was established to be a phenocopy [33]. During 2018, we updated the clinical evaluation of the 41 relatives in generation IV to verify the surprisingly low frequency of affected family members now generally above the risk age (present ages ranging from 46 to 85 years). Of these 41 family members 4 died: 2 from histologically verified HDLS (case 1 [IV:33] with neuropathology reported here, and case 2 [IV:36], with neuropathology reported before [33]); 1 allegedly from complications associated with diabetes (IV-5); and 1 from probable suicide in the context of drug abuse (IV-9). For 23 of the 37 family members currently alive, we identified no symptoms or signs of ongoing HDLS disease by personal contact during 2018 usually supported by formal neurological examination performed by the authors CS and OA. Additionally, no symptoms of HDLS were recorded for the remaining healthy family members taking part in this study as reported by close relatives during 2018. The authors followed 3 of these healthy relatives (IV-34, IV-35 and IV-37, children of HDLS affected individuals), by yearly neurological examinations and cerebral MRI during 5–7 years until 2018, all with negative results. In one individual (IV-38), previous publications and assessments suggested HDLS-S, however no disability developed over time. Even though he declined clinical examination, social function was reported to be excellent, indicating the absence of HDLS. One other family member declined to be contacted but was recently reported by relatives to be healthy.

The present study is based on DNA specimens from the 2 affected and 23 unaffected family members in generation IV that were personally followed by the authors (CS and OA), as indicated in Fig. 1.

### Case 1

Individual IV-33 in Fig. 1, was previously reported [33, 36]. Briefly, at age 46 this previously healthy manual worker suffered a progressive personality change with striking passivity and loss of responsibility at work and at home. At his first examination half a year after onset we observed a debilitating frontal syndrome with total loss of insight, along with discrete pyramidal and deep sensory signs. During the subsequent 2 years he was in a permanent hyperactive state, incessantly walking, opening cupboards or clapping his hands, still with only moderate motor, sensory and extrapyramidal signs. In the fourth year of his disease he had developed complete hemianopia, and showed gradually increasing rigidity in all extremities, as well as primitive brain stem and grasp reflexes. For 6 years he remained in a vegetative state with a general decortical type of rigidity, a weak doll’s eye reaction and spontaneous respiration of Cheyne-Stokes type until he succumbed from respiratory infections at 57 years of age. Five consecutive MRI examinations up to 26 months disease duration with DTI showed a symmetric leukoencephalopathy with an unusual feature, a progressive rim of decreased diffusion expanding centrifugally through the white matter from the periventricular area of the frontal and occipital horns, leaving apparently disorganized tissue behind the rim [36].

### Neuropathology assessment

**Fig. 2:**
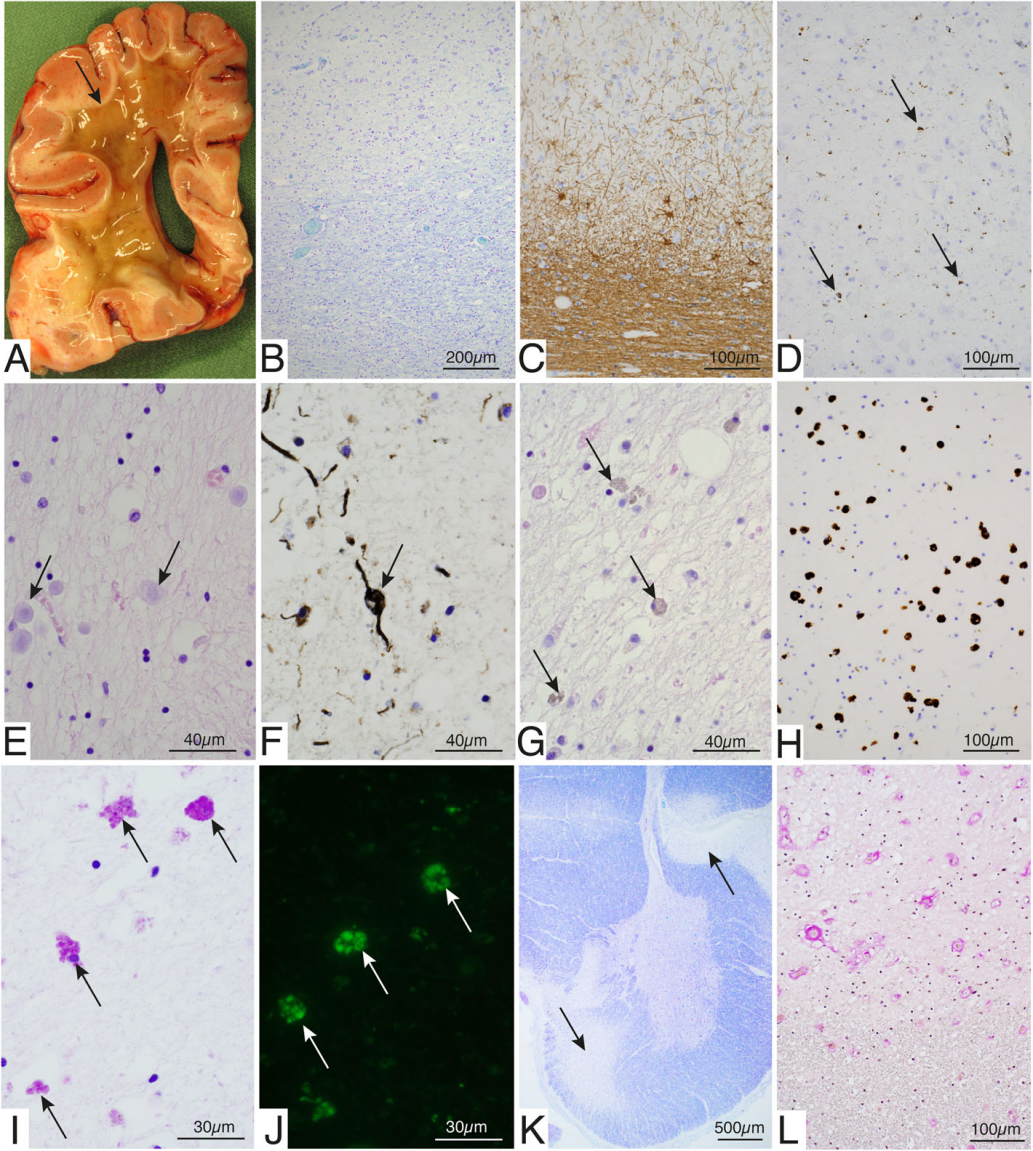
Postmortem examination of brain tissue from Case 1. **a** Transverse section of the brain at the level of the posterior part of the frontal lobe showing liquefaction of the white matter sparing the subcortical arcuate fibers (arrow). **b** Section through the inner part of the cerebral cortex and the subcortical white matter showing preserved cortical neurons and subcortical myelin with some increased cellularity in the white matter (Luxol). **c** Section through the inner part of the cerebral cortex and the subcortical white matter showing slight gliosis of the cortex with preserved neurons and marked gliosis in subcortical white matter. In the transition zone large astrocytes are present (Immunohistochemistry of GFAP). **d** Cerebral cortex showing preserved neurons and some activation of microglial cells (arrows; Immunohistochemistry of CD68). **e**-**h** Sections from the deep white matter just beneath the preserved subcortical parts. **e** There are numerous corpora amylacea (arrows; Hematoxylin and eosin). **f** Fragmented axons with occasional swellings compatible with spheroids in the border (rim region) between preserved cortical white matter and the deep liquified parts (arrow; immunohistochemistry of neurofilament protein). **g** Numerous large rounded pigmented macrophages in the rim (arrows; Hematoxylin and eosin). **h** The rounded large macrophages in the rim region are CD68 positive (Immunohistochemistry of CD68). **i** The macrophages in the rim region contain PAS positive granula (arrows). **j** The storage material in the macrophages is also autofluorescent (arrows). **k** Section from the cervical spinal cord demonstrating marked secondary demyelination of the lateral and anterior corticospinal tracts (arrows, Luxol fast blue-cresyl violet). **l** The anterior corticospinal tract (upper part) showing vascular changes with thickened walls as compared with the adjacent, less affected white matter (lower part) (van Gieson)

The brain weighed 1480 g. The leptomeninges were essentially normal and there was no apparent gyral atrophy. There were only minor atheromatous plaques in the extracerebral arteries on the brain surface. Transverse sections of the cerebrum showed extensive liquefaction of the white matter encompassing the centrum semiovale and surrounding the ventricles but sparing a subcortical rim including the arcuate fibers (Fig. 2a). The frontal parts of the temporal lobes were also spared. Corpus callosum and the internal capsule showed the same gelatinous liquified appearance, as well as the entire white matter surrounding the basal ganglia and the thalamus. The pons was flattened and there was a yellowish discoloration of the basal aspect of the lower brain stem corresponding to the pyramidal tracts. The cerebellum showed minor atrophy of the folia and the spinal cord appeared macroscopically normal.

Microscopic examination of the cerebral cortex and underlying white matter showed essentially the same changes in various parts of the brain (Fig. 2). There was no major loss of cortical neurons but some appeared swollen. There were no neurofibrillary tangles or senile plaques as revealed by ubiquitin immunohistochemistry. There was mild gliosis that was variable in different parts of the brain (Fig. 2c) and microglial activation was also seen in the cortex (Fig. 2d). The subcortical white matter was to some extent spared (Fig. 2a-b) but showed accumulation of macrophages and marked gliosis (Fig. 2c). Occasional spheroids could be identified by hematoxylin and eosin staining and by immunohistochemistry for neurofilament protein, however, they were not abundant. The white matter in the border between the partly spared subcortical white matter and the liquified deep parts, showed increased number of cells and frequent corpora amylacea (Fig. 2e), fragmented axons partly with spheroid-like changes (Fig. 2f) and numerous large rounded pigmented macrophages (Fig. 2g-h) with PAS positive and autofluorescent granula (Fig. 2i-j) indicating storage of lipofuscin. Many macrophages included ubiquitinated inclusions. The deep white matter that had undergone severe degeneration with a gelatinous appearance showed sparse cell nuclei.

The white matter of the internal capsule and the pyramidal tracts of the brain stem showed severe loss of myelin and gliosis. The pyramidal tracts showed vascular changes with thickening of the walls and narrow lumina (see below, spinal cord) but no general vasculopathy was observed in the brain.

The basal ganglia and the thalamus appeared reduced in size and there was gliosis but no major reduction in neuronal cell density. Neither was there a major loss of neurons in the cranial nerve nuclei or the inferior olives. In the cerebellum there was a variable loss of Purkinje cells with a concomitant proliferation of Bergmann glia. There was gliosis in the cerebellar white matter and occasional spheroids, but the neurons of the dentate nucleus appeared to be spared. In the spinal cord there was an asymmetric degeneration of the pyramidal tracts affecting both the lateral and anterior cortico-spinal tracts with vascular changes (Fig. 2j-k). The dorsal columns were much less affected. The neurons of the anterior horns were preserved but frequently swollen. The anterior and posterior nerve roots were preserved as was the sciatic nerve.

### Case 2

Individual IV-36 in Fig. 1, was previously reported [33]. Briefly, at age 34 this woman, who worked with computer programming, had an insidious onset of cognitive problems followed by increasing sensibility disturbance of her right hand, leading to profound deep sensory ataxia difficult to distinguish from alien hand. In parallel she started to present dystonic and ballistic movements in her extremities, soon compromising her gait. Within months she was wheelchair bound with function loss from multiple cerebral regions, including a general pyramidal syndrome, and complete loss of her inferior visual fields. Two years after onset she was in an uncommunicative state, blind, with short attacks of severe rigidity. Her MRI showed symmetrical changes around the frontal and posterior horns, extending into the parietal centrum semiovale and across the corpus callosum, except for its midportion, also following the corticospinal tracts into the mesencephalon. HDLS was confirmed by characteristic autopsy findings as reported [33].

### Genetic analyses

**Fig. 3:**
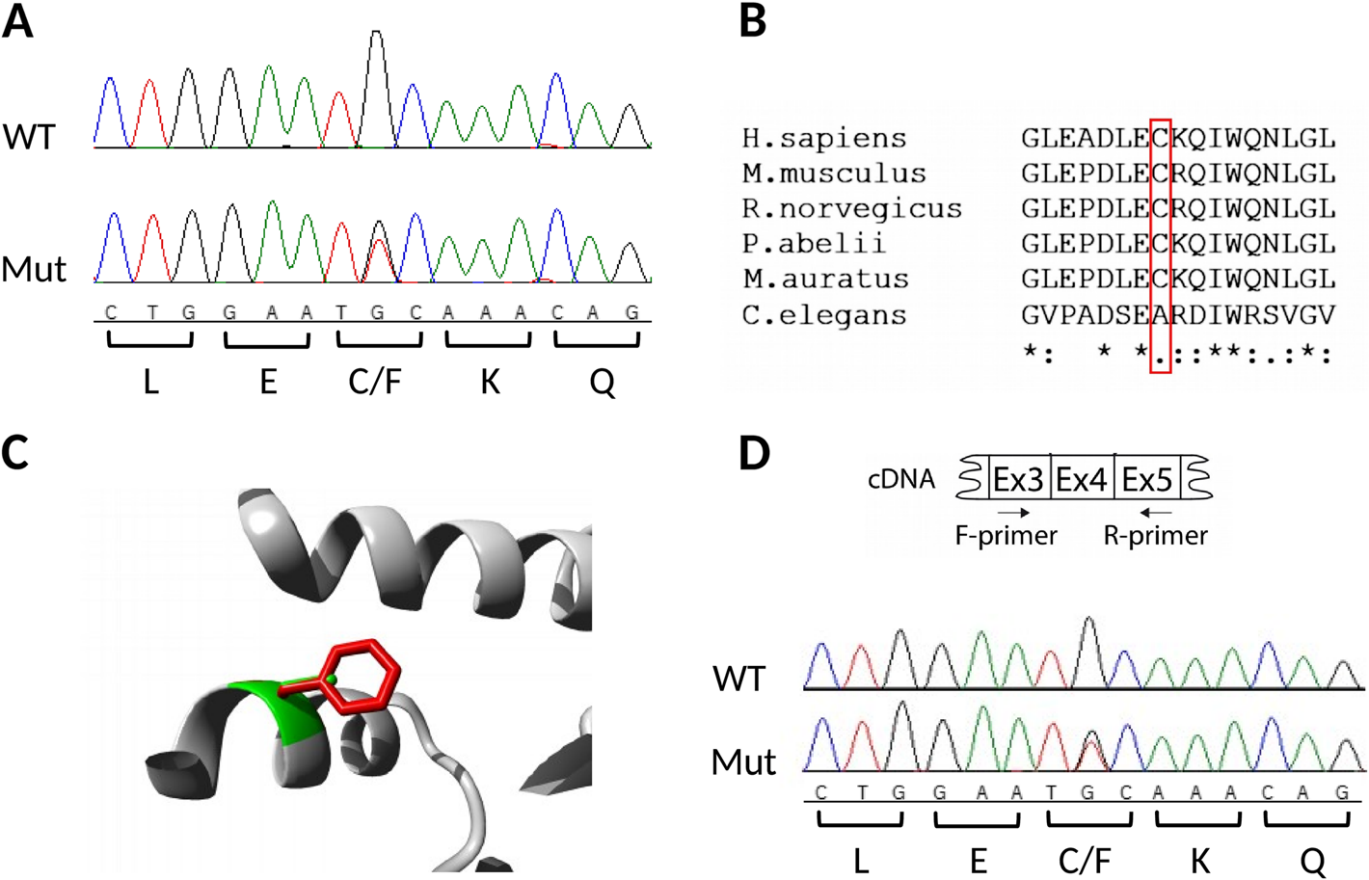
Main features of the *AARS* variant identified in the studied HDLS-S family. **a** Sanger sequence electropherograms showing the wild-type nucleotides present in an unaffected family member (on top) and the heterozygous variant found in both patients (bottom). **b** Multiple-species amino acid sequence alignment of AARS showing the variant region. Position 152, where the variant occurs, is marked with a red box. Species with reviewed sequences in the Uniprot database were selected for the alignment (P49588–1, Q8BGQ7–1, P50475–1, Q5RC02–1, Q8CFX8–1, O01541–1). The alignment was performed using Clustal Omega (https://www.ebi.ac.uk/Tools/msa/clustalo/). An ‘*’ (asterisk) on the bottom of the alignment indicates positions which have a single, fully conserved residue; a ‘:’ (colon) indicates conservation between groups of strongly similar properties; and a ‘.’ (period) indicates conservation between groups of weakly similar properties. **c** Comparison of the side chain of the wild-type Cysteine, a polar amino acid with a non-charged residue (green), and the larger aromatic side chain of the mutant, hydrophobic Phenylalanine (red). The image was obtained from the PDB structure 4XEM using the HOPE tool (http://www.cmbi.ru.nl/hope/about/). **d** Sanger sequence electropherograms after cDNA synthesis showing no splicing changes and similar expression of both alleles in occipital brain tissue of case 1 (bottom)

Previous Sanger sequencing of *CSF1R* coding region in both patients did not identify rare variants predicted to be damaging and that could explain the disease in this family (data not shown). All variants identified in *CSF1R* by exome sequencing are presented in Additional file 1: Table S1. The analysis of genotyping array data from the *CSF1R* locus did not reveal any copy number variants (CNVs) that could be involved in the disease either. Similarly, the same analysis at a genome level did not identify CNVs shared by the two affected individuals and that could be considered pathogenic. Exome sequencing analysis revealed that both patients carried variants that were novel, heterozygous, and predicted to be deleterious in two genes associated with the nervous system: NM_001605.2:c.455G > T p.(Cys152Phe) in *AARS* and NM_024939.3:c.728 T > G p.(Val243Gly) in *ESRP2*. Both variants were absent in the two healthy relatives. After segregation analysis to validate the association of the variants with the disease, only p.Cys152Phe in *AARS* remained absent in all the healthy family members tested (Fig. 1 and Additional file 1: Figure S1). This variant is not described in the genome aggregation database (gnomAD), and it was not found in 1000 Swedish individuals [3]. The variant was predicted as damaging by different software: SIFT, PolyPhen-2 and MutationTaster2, and has a high CADD score of 34, suggesting a deleterious effect of the variant in the protein function. It occurs in an evolutionarily conserved amino acid located in the aminoacylation domain of the protein (Fig. 3). Expression analysis at transcript level revealed that both alleles are expressed equally and that no splice defect could be seen (Fig. 3d).

## Discussion

Hereditary Diffuse Leukoencephalopathy with Spheroids of the Swedish type (HDLS-S) is a devastating adult-onset leukoencephalopathy. Its histopathology supports its relationship to neuraxonal degenerations, a group currently breaking up in several nosographic units defined by genetics. Here we demonstrate that the p.Cys152Phe variant in *AARS* is the likely cause of HDLS-S, thus separating this nosographic entity from *CSFR1R* related HDLS/ALSP. At the present time, no systematic studies have yet distinguished the phenotypes of HDLS/ALSP and HDLS-S. Both have adult onset, with average age at HDLS/ALSP onset of 43 years and average age at onset for HDLS-S of 40; and average age at death of 53 and 55 years, respectively. Both diseases present an insidious course with an initial frontal lobe or cognitive syndrome of varying duration developing into a variable focal, multisystem encephalopathy, where reported Parkinsonian features in the CSF1R-related HDLS/ALSP cases may correspond to extrapyramidal and dystonic features in HDLS-S [4, 14, 33–35]. However, recently reported MRI characteristics may be unique for HDLS-S [36]. Results from the postmortem examination of the brain apparently represented a late outcome of the disease after many years of vegetative state. The white matter was nearly completely liquified in the cerebrum and pyramidal tracts but some regions such as the subcortical white matter, the cerebellum and ascending tracts in the spinal cord were less affected. In a previous study on this patient (case 1 reported here) based on five consecutive MRI examinations performed 10 to 26 months after symptom onset, diffusion tensor imaging (DTI) sequences showed that a centrifugally expanding front of diffusion abnormalities appeared around the anterior ventricular horns [36]. Eventually, similar peripherally progressing changes occurred in the posterior white matter. The rim showed marked restriction of diffusivity suggestive of hypercellularity, and behind the rim was an enlarging center of marked increase in both axial and radial diffusivity indicating general tissue destruction and extensive damage to white matter integrity. At the time of the last MRI examination, 7 years before death, the patient was in the hyperactive stage. We performed no further imaging tests during the subsequent, extremely protracted, terminal stages. The results from the postmortem investigation demonstrated that there was increased cellularity including astrocytes and numerous large lipofuscin containing macrophages in the rim region. We suggest that the hypercellular rim identified by DTI may have expanded slowly further towards the cortex, and that it anatomically corresponds to the preserved subcortical white matter with increased cellularity demonstrated histologically (Fig. 2).

**Fig. 4:**
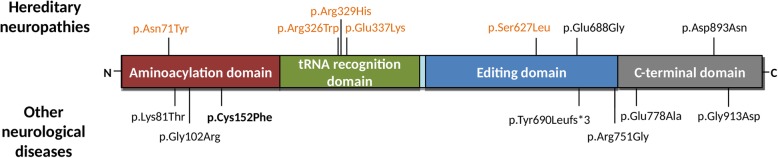
Schematic representation of AARS (NP_001596) with the variants previously identified in hereditary neuropathies (top) and other neurological diseases (bottom) represented. Only variants with a clear association with disease and present in Table 1 are represented here. Variants associated with axonal CMT2 are represented in orange. The variant p.Cys152Phe found in this study is represented in bold. The distribution of variants along the protein shows there is no relation between the location of variants with the associated phenotype. Other variants identified in the gene but with less information are summarized in Additional file 1: Table S2. All variants associated with hereditary neuropathies were found in the heterozygous state. Mutations identified in other neurological disorders were found in the homozygous (p.Arg751Gly), compound heterozygous (p.Tyr690Leufs*3;p.Gly913Asp and p.Lys81Thr;p.Arg751Gly) and heterozygous (p.Gly102Arg) states

For some years, the decisive dividing criterion between HDLS/ALSP and HDLS-S remained the negative findings of *CSF1R* mutations in HDLS-S. Here we report that a novel heterozygous p.Cys152Phe variant in *AARS* is the most likely cause of HDLS-S. *AARS* encodes the alanyl-tRNA synthetase, responsible for the attachment of alanine to its cognate tRNA, in the first step of protein translation. This enzyme has two important domains where the majority of disease-associated mutations are located: the aminoacylation domain responsible for the ligation of the amino acid to tRNA, and the editing domain that hydrolyses mischarged tRNA [10]. Dominant mutations in this gene have been described as affecting the peripheral nervous system causing Charcot-Marie-Tooth disease (CMT) type 2 [16], other hereditary neuropathies, and an indolently progressive, mild form of myeloneuropathy [24]. Axonal degeneration is a common feature for HDLS-S and CMT2N, however no signs of neurodegeneration were observed in the peripheral nervous system specimens (nerve roots and sciatic nerves) from the HDLS-S case 1 examined here. The cellular location of the enzyme is in the perikaryon (Nissl substance), with a very high demand for protein synthesis for the axon, but also in dendrites and spines. However, studies on the relationship between disturbed protein biosynthesis or modification in the perikaryon and specific structures in peripheral nerves are still immature [40]. Furthermore, bi-allelic mutations in *AARS* have also been associated with more severe recessive early-onset epileptic encephalopathies with hypomyelination [25, 31]. The exact mechanism(s) linking the mutations to the different phenotypes is still unknown. The mutations identified so far are distributed equally across the protein without a clear correlation with the different phenotypes (Fig. 4) and the majority decreases the aminoacylation activity (p.Asn71Tyr, p.Lys81Thr, p.Arg329His, p.Tyr690Leufs*3, p.Arg751Gly and p.Gly913Asp), or the editing activity (p.Tyr690Leufs*3) of the protein (Table 1). However, p.Glu337Lys was described to be a gain-of-function mutation, increasing the catalytic efficiency of aminoacylation and leading to an aberrant morphology and neurologic phenotype in zebrafish [41]. Reduced protein expression (p.Tyr690Leufs*3 and p.Gly913Asp) [25] or formation of aggregates (p.Asn71Tyr) [37] have also been observed. The p.Cys152Phe described here is located in the aminoacylation domain, suggesting a functional impact in the protein aminoacylation activity. Expression analysis at transcript level showed no splicing alterations and the expression of both alleles in brain tissue. Experimental studies are necessary to evaluate the specific consequences of this variant to protein function.

**Table 1:** Summary of the most informative reports describing mutations in *AARS*

Variant	Zygosity	Cases/Families	Diagnosis, clinical features	Age of onset	Functional studies	Gain/loss of function	References
p.Asn71Tyr c.211A > T	HT	1 Taiwanese family	CMT2 ^a^ Slowly progressing sensorimotor LE and UE neuropathy NCV: intermediate^b^	11-45y	Not localized in cytoplasm but in organelles, formation of aggregates, growth inhibition, aminoacylation severely defective		[37] [23] [19]
p.Lys81Thr c.242A > C	compound HT^c^	2 mixed-European descent siblings	Microcephaly, epileptic encephalopathy with persistent myelination defect	Congenital, 3mo	Aminoacylation mildly defective, reduced yeast cell survival	LoF	[31]
p.Gly102Arg c304G > C	HT	1 North American family	Mild axonal neuropathy with hyperreflexia indicating superimposed myelopathy NCV: Normal or slight decrease	Third to fifth decade of life	No yeast cell growth	LoF	[24]
p.Arg326Trp c.976GC > T	HT	1 Dutch family	CMT2N Severe motor (sensorimotor) LE polyneuropathy with axonal and demyelinating, or pure axonal features NCV: intermediate or normal	First to third decade of life	No yeast cell growth, toxicity in zebrafish embryos, with aberrant morphology and neurologic phenotype	LoF	[41]
p.Arg329His c.986G > A	HT	2 French families	CMT2 Sensorimotor LE or LE and UE axonal neuropathy, NCV: slight to moderate decrease	6-54y		LoF	[14]
		1 Australian family	CMT2N Axonal sensorimotor neuropathy and variable sensorineural deafness NCV: intermediate	Early onset	Aminoacylation severely defective		[23]
		5 British families	CMT2 sensorimotor poly- or LE neuropathy NCV: intermediate	Congenital to 30y			[5]
p.Glu337Lys c.1009G > A	HT	1 family	CMT2 Severe sensorimotor polyneuropathy with axonal and demyelinating features NCV: moderate decrease	First to third decade of life	Increased yeast growth, increased aminoacylation efficiency, toxicity in zebrafish embryos, with aberrant morphology and neurologic phenotype	GoF	[41]
p.Ser627Leu c.1880C > T	HT	1 family	CMT2N Severe axonal sensorimotor LE and UE neuropathy, predominantly distal NCV: intermediate	Third decade of life	Reduced yeast viability; toxicity in zebrafish embryos, with aberrant morphology and neurologic phenotype	LoF	[41]
p.Glu688Gly c.2063A > G	HT	1 British family	CMT2 pedigree with transitional forms to CMT1 sensorimotor poly- or LE neuropathy; split hand deformity NCV: intermediate	Congenital to first decade of life			[5]
p.Tyr690Leufs^*^3 c.2067dupC	compound HT^d^	2 mixed-European descent sisters	Progressive microcephaly, MRI shows hypomyelination; epileptic encephalopathy; spastic paraplegia	Congenital	Reduced protein expression, reduced aminoacylation activity, reduced editing activity, accumulation of [3H]Ser-tRNA Ala	LoF	[25]
p.Arg751Gly c.2251A > G	compound HT^c^, HM	1 mixed-European descent individual	Microcephaly, epileptic encephalopathy with persistent myelination defect	Congenital, 3mo	Aminoacylation severely defective, normal editing activity, normal yeast survival	LoF	[27]
p.Glu778Ala c.2333A > C	HT	1 Australian family	Possible CMT2N Rippling muscles, cramps, and polyneuropathy; or only rippling muscles and cramps NCV: severe axonal lesions	N.A.	Normal yeast growth, normal localization, normal aminoacylation, normal editing activity.		[23]
p.Asp893Asn	HT	1 Chinese family	Distal hereditary motor neuropathy (dHMN) Mild UE weakness, slow progression, no sensibility disturbance. Normal NCV, but EMG with neurogenic lesion	First to sixth decade of life			[42]
p.Gly913Asp c.2738G > A	compound HT^d^	2 mixed-European descent sisters	Progressive microcephaly, hypomyelination, epileptic encephalopathy, spastic paraplegia	Congenital	Reduced protein expression, reduced aminoacylation activity, normal editing activity		[25]

Other variants identified in the gene but associated with less clinical information or without a confirmed association with disease are presented in Additional file 1. Many CMT2 reports include statements that genes most frequently implicated in CMT were excluded. *CMT* Charcot-Marie-Tooth disease, *CMT2N* Charcot-Marie-Tooth disease type 2 N, *HT* heterozygous mutation, *HM* homozygous mutation, *LoF* loss-of-function mutation, *GoF* gain-of-function mutation, *NCV* nerve conduction velocity, *UE* upper extremity, *LE* lower extremity, *EMG* electromyography. ^a^According to the European CMT consortium diagnostic guidelines ^b^Intermediate NCV defined to be 25–45 m/sec. ^c^ Compound heterozygous sample NP_001596:p.[Lys81Thr];[p.Arg751Gly] ^d^ Compound heterozygous sample NP_001596:p.[Tyr690Leufs*3];p.[Gly913Asp]

Lee et al. (2006) showed that sticky (sti) mutant mice carrying a point mutation (p.Ala734Glu) in the editing domain of AlaRS have ubiquitinated protein aggregates in cerebellar Purkinje cells, resulting in the degeneration of these neurons and ataxia [17]. This missense mutation was located in the editing domain of the protein and was shown to compromise the proofreading activity of this enzyme during aminoacylation of tRNAs. These findings provided a novel mechanism underlying neurodegeneration, caused by accumulation of misfolded proteins and cell death resulting from the disruption of translational fidelity in terminally differentiated neurons.

Mutations in other tRNA synthetases have also been associated with leukoencephalopathy. Several cases of adult-onset leukodystrophy were recently shown to be caused by bi-allelic mutations in *AARS2*, the gene encoding the mitochondrial alanyl-tRNA synthetase, expanding the spectrum of adult onset neuroaxonal degeneration disorders [6, 7, 20, 28]. The clinical phenotypes of *CSF1R* and *AARS2* related ALSPs showed minor differences [15], sharing major clinical, imaging, and pathologic features, such as cognitive and motor symptoms [20]. While the histological hallmark of neuraxial degeneration with spheroids is common to the three disorders: *AARS2* leukoencephalopathy, *CSF1R* related HDLS/ALSP, and HDLS-S (with the latter two expected to be most closely related), some differences have been observed, including an earlier age of onset of leukodystrophy (third decade of life) in addition to ovarian failure associated to *AARS2* mutations [15]. Systematic comparison of MRI phenotypes showed more brain stem and deeper gray matter lesions in *AARS2* disease [15]. Early white matter lesions may be more symmetric in HDLS-S, and the centrifugal periventricular rim of low diffusion observed in case 1 [33, 36] was not observed in HDLS/ALSP or in AARS2, although similar consecutive DTI studies were not performed in these diseases [7]. Small cerebral calcifications with stepping-stone appearance were described in HDLS/ALSP; the present investigation did not include high-resolution computerized tomography required to search for such calcifications [14].

The frequency of HDLS-S cases in generation IV is unexpectedly low, given the previous assessments of the family. The number of well-documented cases in family members of generations II, III and IV were 4/9, 4/24 and 2/41 (Fig. 1). In families with high degrees of organic psychiatric morbidity, including suicides, it may be difficult to track offspring. However, the segregation pattern in the geographically less scattered descendants of II:9 followed and analyzed at our department, comprising cases 1 and 2 in the present study, is compatible with a dominant pattern of inheritance and high penetrance.

## Conclusions

A novel p.Cys152Phe variant in *AARS* is the most likely genetic cause of HDLS-S, a severe adult-onset leukoencephalopathy with the histopathological feature of neuroaxonal degeneration with spheroids. This variant is located in the aminoacylation domain of the protein, but the molecular effects of the variant are still unknown. This is hitherto the only reported family with HDLS-S, but increasing knowledge of relevant mutations will facilitate diagnostic clarification of the group of leukoencephalopathies with unknown etiology.

## Supplementary information

**Additional file 1.** Additional details for Materials and methods and Results.

## Data Availability

All data generated or analyzed during the current study are available from the corresponding authors on reasonable requests.

## References

[1] Adams SJ, Kirk A, Auer RN (2018). Adult-onset leukoencephalopathy with axonal spheroids and pigmented glia (ALSP): integrating the literature on hereditary diffuse leukoencephalopathy with spheroids (HDLS) and pigmentary orthochromatic leukodystrophy (POLD). J Clin Neurosci.

[2] Adzhubei IA (2010). A method and server for predicting damaging missense mutations. Nat Methods.

[3] Ameur A (2017). SweGen: a whole-genome data resource of genetic variability in a cross-section of the Swedish population. Eur J Hum Genet.

[4] Axelsson R (1984). Hereditary diffuse leucoencephalopathy with spheroids. Acta Psychiatr Scand Suppl.

[5] Bansagi B (2015). Genotype/phenotype correlations in AARS-related neuropathy in a cohort of patients from the United Kingdom and Ireland. J Neurol.

[6] Carle G (2018). Alanyl-tRNA Synthetase 2-related dementia with selective bilateral frontal cystic Leukoencephalopathy. J Clin Neurol.

[7] Dallabona C (2014). Novel (ovario) leukodystrophy related to AARS2 mutations. Neurology.

[8] DePristo MA (2011). A framework for variation discovery and genotyping using next-generation DNA sequencing data. Nat Genet.

[9] Guerreiro R (2013). Genetic analysis of inherited leukodystrophies: genotype-phenotype correlations in the CSF1R gene. JAMA Neurol.

[10] Guo M (2009). The C-Ala domain brings together editing and aminoacylation functions on one tRNA. Science.

[11] Hakola HP, Jarvi OH, Sourander P (1970). Osteodysplasia polycystica hereditaria combined with sclerosing leucoencephalopathy, a new entity of the dementia praesenilis group. Acta Neurol Scand.

[12] Kircher M (2014). A general framework for estimating the relative pathogenicity of human genetic variants. Nat Genet.

[13] Kohler W, Curiel J, Vanderver A (2018). Adulthood leukodystrophies. Nat Rev Neurol.

[14] Konno T (2017). Clinical and genetic characterization of adult-onset leukoencephalopathy with axonal spheroids and pigmented glia associated with CSF1R mutation. Eur J Neurol.

[15] Lakshmanan R (2017). Redefining the phenotype of ALSP and AARS2 mutation-related leukodystrophy. Neurol Genet.

[16] Latour P (2010). A major determinant for binding and aminoacylation of tRNA (Ala) in cytoplasmic Alanyl-tRNA synthetase is mutated in dominant axonal Charcot-Marie-tooth disease. Am J Hum Genet.

[17] Lee JW (2006). Editing-defective tRNA synthetase causes protein misfolding and neurodegeneration. Nature.

[18] Li H, Durbin R (2010). Fast and accurate long-read alignment with burrows-wheeler transform. Bioinformatics.

[19] Lin KP (2011). The mutational spectrum in a cohort of Charcot-Marie-tooth disease type 2 among the Han Chinese in Taiwan. PLoS One.

[20] Lynch DS (2016). Analysis of mutations in AARS2 in a series of CSF1R-negative patients with adult-onset Leukoencephalopathy with axonal spheroids and pigmented glia. JAMA Neurol.

[21] Lynch DS (2017). Clinical and genetic characterization of leukoencephalopathies in adults. Brain.

[22] McKenna A (2010). The genome analysis toolkit: a MapReduce framework for analyzing next-generation DNA sequencing data. Genome Res.

[23] McLaughlin HM (2012). A recurrent loss-of-function alanyl-tRNA synthetase (AARS) mutation in patients with Charcot-Marie-tooth disease type 2N (CMT2N). Hum Mutat.

[24] Motley WW (2015). A novel AARS mutation in a family with dominant myeloneuropathy. Neurology.

[25] Nakayama T (2017). Deficient activity of alanyl-tRNA synthetase underlies an autosomal recessive syndrome of progressive microcephaly, hypomyelination, and epileptic encephalopathy. Hum Mutat.

[26] Peiffer J (1977). Generalized giant axonal neuropathy: a filament-forming disease of neuronal, endothelial, glial, and schwann cells in a patient without kinky hair. Acta Neuropathol.

[27] Rademakers R (2011). Mutations in the colony stimulating factor 1 receptor (CSF1R) gene cause hereditary diffuse leukoencephalopathy with spheroids. Nat Genet.

[28] Schiffmann R (1997). Leukodystrophy in patients with ovarian dysgenesis. Ann Neurol.

[29] Schwarz JM (2014). MutationTaster2: mutation prediction for the deep-sequencing age. Nat Methods.

[30] Sim NL (2012). SIFT web server: predicting effects of amino acid substitutions on proteins. Nucleic Acids Res.

[31] Simons C (2015). Loss-of-function alanyl-tRNA synthetase mutations cause an autosomal-recessive early-onset epileptic encephalopathy with persistent myelination defect. Am J Hum Genet.

[32] Smedley D (2015). Next-generation diagnostics and disease-gene discovery with the exomiser. Nat Protoc.

[33] Sundal C (2012). Update of the original HDLS kindred: divergent clinical courses. Acta Neurol Scand.

[34] Sundal C (2012). Hereditary diffuse leukoencephalopathy with axonal spheroids (HDLS): a misdiagnosed disease entity. J Neurol Sci.

[35] Sundal C (2013). Parkinsonian features in hereditary diffuse leukoencephalopathy with spheroids (HDLS) and CSF1R mutations. Parkinsonism Relat Disord.

[36] Sundal C (2014). Different stages of white matter changes in the original HDLS family revealed by advanced MRI techniques. J Neuroimaging.

[37] Tatsumi Y (2019). CMT type 2N disease-associated AARS mutant inhibits neurite growth that can be reversed by valproic acid. Neurosci Res.

[38] Van Bogaert L, Nyssen R (1936). Le type tardif de la leukodystrophie progressive familiale. Rev Neurol.

[39] van der Knaap MS (2000). Autosomal dominant diffuse leukoencephalopathy with neuroaxonal spheroids. Neurology.

[40] Weis J (2017). Towards a functional pathology of hereditary neuropathies. Acta Neuropathol.

[41] Weterman MAJ (2018). Hypermorphic and hypomorphic AARS alleles in patients with CMT2N expand clinical and molecular heterogeneities. Hum Mol Genet.

[42] Zhao Z (2012). Alanyl-tRNA synthetase mutation in a family with dominant distal hereditary motor neuropathy. Neurology.

