# Supplementary material for: An AARS variant as the likely cause of Swedish type hereditary diffuse leukoencephalopathy with spheroids
Source: Acta Neuropathol Commun. 2019 Nov 27;7:188. doi: 10.1186/s40478-019-0843-y (PMC6880494; doi:10.1186/s40478-019-0843-y)
Supplement: Supplementary file 1 — Additional file 1. Additional details for Materials and methods and Results. [file 40478_2019_843_MOESM1_ESM.docx]

Additional file

An *AARS* variant as the likely cause of Swedish type hereditary diffuse leukoencephalopathy with spheroids

Christina Sundal^1^, Susana Carmona^2^, Maria Yhr^3^, Odd Almström^1^, Maria Ljungberg^4^, John Hardy^5^, Carola Hedberg-Oldfors^3^, Åsa Fred ^6^, José Brás^2,7^, Anders Oldfors^3^, Oluf Andersen^1*#^, Rita Guerreiro^2,7*#^

1. Department of Clinical Neurology, Institute of Neuroscience and Physiology, the Sahlgrenska Academy, University of Gothenburg, Göteborg, Sweden
2. Center for Neurodegenerative Science, Van Andel Research Institute, Grand Rapids, Michigan, USA
3. Department of Laboratory Medicine, Institute of Biomedicine, the Sahlgrenska Academy, University of Gothenburg, Göteborg, Sweden
4. Department of Radiation Physics, Institute of Clinical Sciences, the Sahlgrenska Academy, University of Gothenburg, Göteborg, Sweden.
5. Department of Neurodegenerative Disease, Reta Lila Weston Laboratories, Queen Square Genomics, UCL Dementia Research Institute, London, UK
6. Department of Pathology, Hospital of Halland, Halmstad, Sweden
7. Division of Psychiatry and Behavioral Medicine, Michigan State University College of Human Medicine, Grand Rapids, MI, USA.

* These authors contributed equally to this work.

#Correspondence to:

Oluf Andersen

Department of Clinical Neurology, Institute of Neuroscience and Physiology, The Sahlgrenska Academy, University of Gothenburg, Göteborg, Sweden. Address: Neurology, Gröna Stråket 11, 3^rd^ floor, Sahlgrenska University Hospital, 413 45 Göteborg, Sweden.

E-mail: [oluf.andersen@neuro.gu.se](mailto:oluf.andersen@neuro.gu.se)

Rita Guerreiro

Center for Neurodegenerative Science, Van Andel Research Institute, 333 Bostwick Ave. N.E., Grand Rapids, Michigan 49503-2518, USA

E-mail: rita.guerreiro@vai.org

***In silico analysis of the exome sequencing data:***

The variants identified by exome sequencing were ranked using Exomiser. In parallel we used a variety of filtering approaches to narrow down the list of potential variants and genes. First, single nucleotide polymorphisms (SNPs) and insertions and deletions (Indels) were filtered to select exonic and splice-site variants. With the second filter, synonymous variants were excluded from the analysis. After this, variants with a minor allele frequency (MAF) higher than 0.1% in gnomAD database were also excluded.

Given that we are analyzing one family we expect the affected family members to share the same causative variant. Accordingly, the next filtering step involved the selection of variants shared between the two affected individuals. Even though the family pedigree indicated an apparent autosomal dominant mode of inheritance we have also tested the possibility of disease segregation through an autosomal recessive mode of inheritance and two different strategies were applied according to the inheritance pattern: 1) when assuming an autosomal dominant pattern we kept heterozygous variants that were identified in both cases and absent in unaffected individuals; 2) when assuming an autosomal recessive pattern, we kept homozygous or two heterozygous variants in the same gene (as a proxy for compound heterozygosity) that were shared by the two affected individuals and were either absent in the unaffected individuals, were heterozygous in the unaffected subjects or only one of the two variants found to be potentially compound heterozygous were present in the unaffected family members. The predicted functional impact of the variants was evaluated using the *in silico* tools SIFT, PolyPhen-2, MutationTaster and CADD. Gene function and possible associated diseases were evaluated using Genecards and OMIM databases in addition to relevant literature research.

**Figure S1. Sanger sequencing results for the *AARS* locus harboring the p.Cys152Phe in the 25 family members tested.** Identification of samples according to the pedigree (Figure 1).s


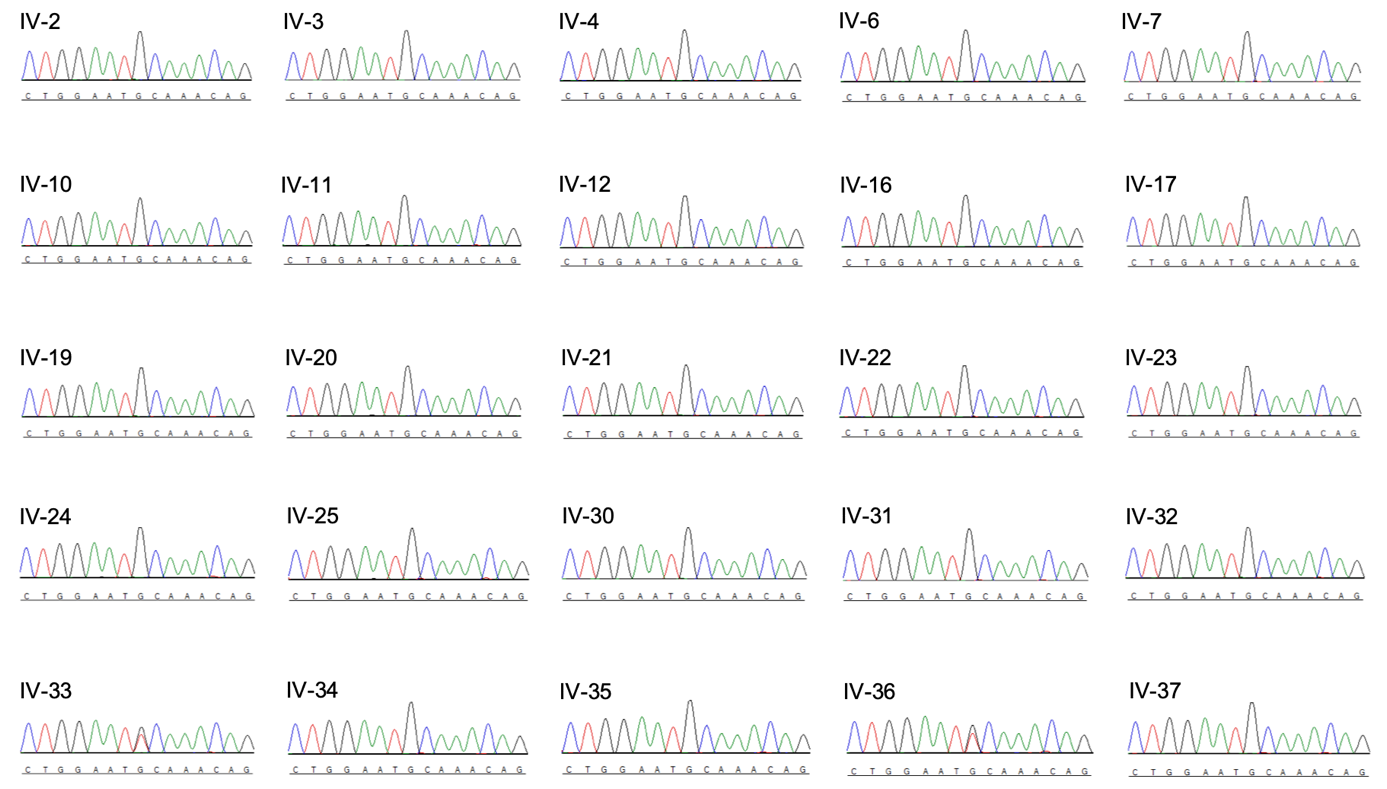


**Table S1. *CSF1R* variants identified by exome sequencing in the two affected and two unaffected family members.**

| **Chr** | **Position** | **rsID** | **Ref** | **Alt** | **Type of variant** | **Consequence** | **MAF (gnomAD NFE)** | **MAF (gnomAD Finnish)** | **IV-21** | **IV-33** | **IV-35** | **IV-36** |
| --- | --- | --- | --- | --- | --- | --- | --- | --- | --- | --- | --- | --- |
| **5** | 149433400 | rs3216780 | G | GC | 3_prime_UTR | c.*231_*232insG | 0.6334 | 0.6765 | +/+ | +/+ | +/+ | +/+ |
| **5** | 149433596 | rs2066934 | T | G | 3_prime_UTR | c.*36A>C | 0.7644 | 0.8033 | +/+ | +/+ | +/+ | +/+ |
| **5** | 149433597 | rs2066933 | G | A | 3_prime_UTR | c.*35C>T | 0.7642 | 0.8033 | +/+ | +/+ | +/+ | +/+ |
| **5** | 149434954 | rs72832135 | C | T | intron | c.2555-55G>A | 0.0178 | 0.0046 | +/- | -/- | -/- | -/- |
| **5** | 149435759 | rs216136 | G | A | intron | c.2442+23C>T | 0.6051 | 0.6675 | +/+ | +/+ | +/+ | +/+ |
| **5** | 149437190 | rs216138 | C | G | intron | c.2133-35G>C | 0.7601 | 0.7964 | +/+ | +/+ | +/+ | +/+ |
| **5** | 149441016 | rs1259139542 | AC | A | intron | c.1858+37delG | 0.6308 | 0.6988 | +/+ | +/+ | +/+ | +/+ |
| **5** | 149450132 | rs10079250 | T | C | missense_variant&splice_region | c.1085A>G (p.His362Arg) | 0.069 | 0.0214 | +/- | -/- | -/- | -/- |
| **5** | 149456771 | rs2855781 | C | A | intron |  | 0.5258 | 0.6666 | -/- | +/+ | +/+ | +/+ |
| **5** | 149456772 | rs60844779 | T | A | intron |  | 0.5257 | 0.6665 | -/- | +/+ | +/+ | +/+ |
| **5** | 149457678 | rs2228422 | G | A | synonymous | c.726C>T (p.Thr242Thr) | 0.5699 | 0.6975 | +/- | +/+ | +/+ | +/+ |
| **5** | 149460553 | rs216123 | A | G | synonymous | c.84T>C (p.Pro28Pro) | 0.5684 | 0.7009 | +/- | +/+ | +/+ | +/+ |

None of these variants in ENST00000286301 segregated with the disease in the family.

**Table S2. Other variants previously identified in *AARS* in studies of the genetic spectrum of hereditary neuropathies.**

| Variant | Zygosity | Cases/Families | Diagnosis, clinical features | Age of onset | References |
| --- | --- | --- | --- | --- | --- |
| p.Asn71Ser  c.212A>G | HT | 1 possible German individual | CMT2, hereditary motor neuropathy | NA | [1] |
| p.Phe110Leu  c.328T>C | HT | 1 German individual | CMT2 | Childhood | [2] |
| p.Phe175Leu  c.525C>G | HT | 1 individual | CMT2 | 14 | [3] |
| p.Ala302Thr  c.904G>A* | HT | 1 individual from Western  India | CMT2 | 9 | [4] |
| p.Arg320His  c.959G>A | HT | 1 Danish individual | CMT  Sensorimotor polyneuropathy, unclassified. | 74** | [5] |
| p.Asn340Ser  c.1019A>G | NA | 1 possible French individual | NA | NA | [6] |
| p.Ile470Met  c.1410C>G | NA | 1 individual | HMSN2 | 40 | [7] |
| p.Thr608Met  c.1823 C>T^#^ | HT | 1 individual | HMSN1  Thenar atrophy  NCV 40-49 m/s. | 20 | [8] |
| p.Gly681Val  c.2042G>T | HT | 1 individual. Mother is a carrier, but asymptomatic | CMT2 | 33 | [9] |
| p.Ala940Val c.2819C>T | HT | 1 individual. Father has recurrent sensory disturbances in UL and LL | CMT: Reduced motor NCV in left ulnar nerve. Sudden onset of muscle weakness and reduced sensibility in left arm. | 39 | [5] |
| p.Phe958Ser  c.2873T>C | HM | 1 Yemen individual | Severe contractures since birth, motor neuropathy, cognitive delay, seizure history and  progressive secondary microcephaly. | Birth | [2] |

Most of these variants were identified in 1 individual in studies of the genetic spectrum of hereditary neuropathies. Functional studies were not performed to validate their impact in protein function and their association with disease. CMT: Charcot-Marie-Tooth disease. CMT2N: Charcot-Marie-Tooth disease type 2. HMSN1: Hereditary motor and sensory neuropathy type 1. HMSN2: Hereditary motor and sensory neuropathy type 1. HT: Heterozygous mutation. HM: Homozygous mutation. NA: not available. NCV: Nerve conduction velocity. UE: Upper extremity. LE: Lower extremity.* Variant considered of uncertain significance by original study. ** Of note, this age is outside the typical age at onset range for CMT with the incidence of neuropathy being high at these older ages. ^#^This patient also carries the *KIT* p.Glu583Gln in the heterozygous state.

References

1. Dohrn, M.F., et al., *Frequent genes in rare diseases: panel-based next generation sequencing to disclose causal mutations in hereditary neuropathies.* J Neurochem, 2017. **143**(5): p. 507-522.

2. Karakaya, M., et al., *Targeted sequencing with expanded gene profile enables high diagnostic yield in non-5q-spinal muscular atrophies.* Hum Mutat, 2018. **39**(9): p. 1284-1298.

3. Bacquet, J., et al., *Molecular diagnosis of inherited peripheral neuropathies by targeted next-generation sequencing: molecular spectrum delineation.* BMJ Open, 2018. **8**(10): p. e021632.

4. Khadilkar, S.V., et al., *Clinico-Electrophysiological and Genetic Overlaps and Magnetic Resonance Imaging Findings in Charcot-Marie- Tooth Disease: A Pilot Study from Western India.* Ann Indian Acad Neurol, 2017. **20**(4): p. 425-429.

5. Vaeth, S., et al., *Genetic analysis of Charcot-Marie-Tooth disease in Denmark and the implementation of a next generation sequencing platform.* Eur J Med Genet, 2019. **62**(1): p. 1-8.

6. Sevy, A., et al., *Improving molecular diagnosis of distal myopathies by targeted next-generation sequencing.* J Neurol Neurosurg Psychiatry, 2016. **87**(3): p. 340-2.

7. Wang, W., et al., *Target-enrichment sequencing and copy number evaluation in inherited polyneuropathy.* Neurology, 2016. **86**(19): p. 1762-71.

8. Schabhuttl, M., et al., *Whole-exome sequencing in patients with inherited neuropathies: outcome and challenges.* J Neurol, 2014. **261**(5): p. 970-82.

9. Liu, Y., et al., *Targeted Next-Generation Sequencing for Clinical Diagnosis of 561 Mendelian Diseases.* PLoS One, 2015. **10**(8): p. e0133636.
